# How to Translate Time? The Temporal Aspect of Human and Rodent Biology

**DOI:** 10.3389/fneur.2017.00092

**Published:** 2017-03-17

**Authors:** Denes V. Agoston

**Affiliations:** ^1^Department of Anatomy, Physiology and Genetics, Uniformed Services University, Bethesda, MD, USA; ^2^Department of Neuroscience, Karolinska Institutet, Stockholm, Sweden

**Keywords:** translation, species, differences, time, humans, rats, mice

Therapeutic interventions can only be effective when administered during their specific “therapeutic window.” A multitude of clinical trials based on highly successful preclinical studies performed mostly in rodents have failed to translate into similarly successful clinical outcomes (1–4). One potentially important but largely ignored factor contributing to these fiascoes is the different biological timescales of rodents versus humans. Here, I compare the rodent and human timescales of major biological events and show that while the timescales of biochemical processes such as enzyme kinetics might be comparable, more complex biological processes such as gestation, sexual maturation, lifespan, etc., run on vastly different timescales in rodents compared to humans. These comparisons strongly indicate that a “rat hour” or “rat day” is also not equivalent to a “human hour” or “human day”—and *vice versa*—when it comes to clinically relevant complex pathologies, such as sepsis, or inflammation (5).

Rodent models of normal biology and diseases are the backbone of modern biomedical research. Species differences have been documented (6), and our lack of understanding between the timescales of rodent and human physiological and pathological processes has been raised during various scientific meetings. Earlier papers have discussed the time differences between rodents and human (7, 8). However, to our knowledge, this is the first paper that summarizes available data about time differences in normal biological processes between the two species in a comprehensive manner.

Pathological processes, especially in case of acute CNS disorders such as traumatic brain injury or stroke, can change rapidly over time so the therapeutic window can be easily missed. One potentially important, but mostly ignored factor contributing to the failed translations of experimental findings into clinical practice is the different biological timescales of rodents versus humans. The simplest biochemical process, such as enzyme kinetics, is on a similar timescale in rodents and in humans (9–11). However, as complexity of the biological process grows, the differences between the timescales of the two species also grow (Table 1). For example, m/tRNA turnover is ~2.5 times faster in rodents (rat) than in humans, whereas protein turnover is ~10 times faster in rodents (12–15). Basal metabolic rate (BMR) is defined as “the minimal rate of energy expenditure per unit time by endothermic animals at rest” (16). The BMR in rats is 8 W/kg as opposed to 1.25 W/kg in humans; in other words, the BMR is ~6.4 times faster in the rodent. Heart rate is on average ~4.7 times faster in the rat than in the human (260–400 versus 60–80 beats/min), and respiratory rate is ~6.3 times faster in the rat than in the human (75–115 versus 12–18/min).

**Table 1:** **Timelines of basic biological processes in the human and in the rat**.

	Human	Rat^a^	Times faster in rat	One human year ≈ rat days	One human day ≈ rat hours	One human hour ≈ rat minutes
m/tRNA turnover (12, 14, 15)	0.8/day/kg	2/day/kg	2.5			
Protein turnover (12, 13)	1.25/day/kg	12/day/kg	9.6			
Metabolic rate (16)	1.25 W/kg	8 W/kg	6.4			
Heart rate (21)	60–80	260–400	4.7			
Respiratory rate (21)	12–18	75–115	6.3			
Gestation (21, 22)	280 days	21–23 days	12.7	28.7	1.9	4.7
Weaning (8, 23)	180 days	21 days	8.6	42.6	2.8	7
Reaching sexual maturity (8, 23, 24)	4,197 days (11.5 years)	50 days	84	4.3	0.3	0.8
Reaching adulthood (8, 23, 24)	7,300 days (20 years)	210 days	35	10.5	0.7	1.7
Reaching reproductive senescence^b^ (21)	18,615 days (51 years)	532 days (1.6 years)	35	10.4	0.7	1.7
Post-senescence (8, 23)	10,585 days (29 years)	486 days	22	16.8	1.1	2.7
Life span (8, 23)	29,200 days (80 years)	1,095 days (3 years)	26.7	13.7	0.9	2.3

*^a^Rattus norvegicus*.

*^b^Females only*.

Compared to humans, rodents live short and accelerated lives (Table 1). Rats live up to 3 years (1,095 days) (7, 8) compared to humans’ ~80 years (29,200 days) (developed countries average, UN World Population Prospects). Generally speaking, rats live ~27 times faster, meaning that 1 rat day would be around 27 human days, and ~13.5 rat days would be the equivalent of 1 human year. Importantly, the temporal differences between humans and rats vary depending on the phase of life. Gestation is completed within 23 days of conception in rats compared to 280 days in humans, indicating an ~12 times accelerated process. Weaning is completed by 34 days postpartum in rats compared to an average of 180 days in humans, an ~5 times faster process. Rats reach sexual maturity around the age of 50 days, whereas humans reach sexual maturity around the age of 11.5 years (4,197 days). Thus, sexual maturation is ~84 times faster in rats where ~4.3 rat days are the “equivalent” of 1 human year. Adulthood, as determined by full musculoskeletal maturity (i.e., the completed fusion of growth plates) is reached by 210 days of age in rats and around 20 years (7,300 days) in humans. This process is ~35 times faster in rats where 1 human year is roughly the equivalent of 10.5 rat days. Reproductive senescence in humans begins at around 51 years (18,615 days) compared to rats’ 15–20 months (~532 days), indicating a 35 times faster process. During adulthood then, one human year would be the equivalent of 12 rat days. Rats live post-senescence for an average of 16 months (486 days) compared to the human period of 29 years (10,585 days); thus, in this phase of life 1 human year would be roughly equal to 17 rat days.

These differences are not surprising given the striking anatomical differences between the species. The “accelerated development” of the rodent seems obvious if we take into account just the differences in size between the rodent and human bodies (0.025–0.4 kg versus 70 kg, a 2,800 − 175 × difference) or brains (0.5–1.8 g versus 1,300 g, a 2,500 − 722 × differences) between rodents and humans. However, these comparisons suggest that complex pathobiological processes, such as inflammatory responses, may also run on different timescales in rodents and in humans. Indeed, the genomic responses in various inflammatory conditions were ~30–50 times faster in the rodent (mice) than in human (5). The ability to precisely translate time between experimental findings using rodent models and clinical practice is especially critical for conditions such as stroke, myocardial infarction, or traumatic brain injury, where the pathological processes are rapidly changing. The efficacy of pharmacological or other interventions that critically depend on timing, e.g., the “golden hour” for thrombolytic treatments in myocardial infarction or ischemic stroke were determined clinically and not by rodent studies (17, 18). Our lack of understanding about the temporal differences between rodent and human pathological processes has likely contributed to the 100% failure rate of neuroprotective treatments showing efficacy in rodent models of TBI (3, 19, 20). Rodent models have provided critical insights into disease mechanisms and helped to identify drug targets. Their usefulness can be greatly increased if we learn how to translate time between species.

## Summary and Future Directions

Available data indicate that there are substantial differences between rodent and human timescales in normal biological processes. Identifying the differences between the rodent and human timescales is especially important to translate successful experimental treatment into clinical practice in acquired disorders such as stroke or TBI. In the absence of detailed information however, no conversion between the two timescales can be formulated. In order to be able to better “translate time” between experimental and clinical studies, we need to perform more longitudinal studies using comparable outcome measures involving both sexes/genders.

## Author Contributions

The author confirms being the sole contributor of this work and approved it for publication.

## Conflict of Interest Statement

The author declares that the research was conducted in the absence of any commercial or financial relationships that could be construed as a potential conflict of interest.
